# Analysis of the Effects of Prey, Competitors, and Human Activity on the Spatiotemporal Distribution of the Wolverine (*Gulo gulo*) in a Boreal Region of Heilongjiang Province, China

**DOI:** 10.3390/biology14091165

**Published:** 2025-09-01

**Authors:** Yuhan Ma, Xinxue Wang, Binglian Liu, Ruibo Zhou, Dan Ju, Xuyang Ji, Qifan Wang, Lei Liu, Xinxin Liu, Zidong Zhang

**Affiliations:** 1 College of Wildlife and Protected Area, Northeast Forestry University, Harbin 150040, China; 2 College of Life Science and Technology, Mudanjiang Normal University, Mudanjiang 157011, China; 3 Wildlife Institute of Heilongjiang Province, Harbin 150081, China; 4 College of Life Science and Technology, Harbin Normal University, Harbin 150025, China; 5 Liaoning Wildlife Protection and Epidemic Disease Monitoring Center, Dalian 116013, China

**Keywords:** spatiotemporal distribution, camera trapping, occupancy model, kernel density estimation

## Abstract

Understanding how endangered carnivores partition spatiotemporal distribution in human-dominated landscapes is pivotal for mitigating biodiversity loss in climate-sensitive boreal ecosystems. Here, we used kernel density data derived from a 16-month camera-trap survey (140 UVL7 cameras), cold single-season (November–April) occupancy models, and MaxEnt 3.4.4 to identify the effects of biotic interactions, anthropogenic disturbance, and environmental factors on the spatiotemporal distribution of the wolverine (*Gulo gulo*) in Beijicun National Nature Reserve, Heilongjiang Province, China. We found that wolverines exhibited crepuscular activity patterns using night-time relative abundance index (NRAI) = 50.29% with bimodal peaks (05:00–07:00, 13:00–15:00), with dawn activity predominant during the warm season (05:00–06:00) and a bimodal activity pattern in the cold season (08:00–09:00, 14:00–15:00). Temporal overlap with prey (overlap coefficient Δ = 0.84) and competitors (Δ = 0.70) was high, but overlap with human-dominated temporal patterns was low (Δ = 0.58). Wolverines avoided human settlements and major roads, preferred moving along forest trails and gentle slopes, and avoided high-altitude deciduous forests. Populations were mainly concentrated in southern Hedong and Qianshao Forest Farms, which are characterized by high habitat integrity, high prey densities, and minimal anthropogenic disturbance. These findings suggest that wolverines may influence boreal trophic networks, especially in areas with intact prey communities, competitors, and spatial refugia from human disturbances. We recommend that habitat protection and management within the natural reserve be prioritized and that sustainable management practices for prey species be implemented to ensure the long-term survival of wolverines.

## Simple Summary

We investigated the spatiotemporal distribution of wolverines and the factors shaping it in Beijicun National Nature Reserve, Heilongjiang Province, China, using infrared camera trapping. Our findings revealed that the spatiotemporal distribution of wolverines is primarily affected by interspecific interactions and anthropogenic disturbances. Although wolverines exhibited high spatial and temporal overlap with both prey and competitors, they appeared to avoid areas with high levels of human activity and were most active at times when human activity was low. “Deciduous broadleaf forest”, “deciduous coniferous forest”, and “slope” were important environmental factors that affected the spatial distribution of wolverines. These results provide critical insights with implications for the development of science-based conservation strategies and management plans for wolverines.

## 1. Introduction

The spatiotemporal distributions of animals are the result of adaptation under different environmental conditions over long periods, and identifying the factors shaping the spatiotemporal distributions of species is a major goal of animal biology [1]. Studies of the spatiotemporal distributions of mammals were initiated in the 1940s and early 1960s [2,3]. Characterizing activity patterns has been a major focus of research on the temporal distributions of animal species [4]. In recent years, the activity patterns of various species have been characterized using kernel density estimation of data derived from infrared camera traps [5,6]. Studies on the temporal distributions of animals have examined daily activity patterns both within and among species, such as predator–prey overlap, as well as competition and coexistence dynamics among sympatric species. These investigations are pivotal for advancing our understanding of animal behavior [7,8].

Species distribution models (SDMs) have become essential tools for exploring the spatial distributions of large and medium-sized endangered carnivores over the past three decades [9,10]. Key models include physiological-based species distribution models [11], null models [12], maximum entropy models (MaxEnt) [13], and occupancy models [14]. Occupancy models are widely used because of their ability to analyze spatial distributions with relatively simple data inputs. Similarly, MaxEnt has become increasingly popular for studies of spatial distributions and habitat suitability because of its high accuracy, even with limited occurrence records or training data, as well as its user-friendly interface [15]. Numerous studies have demonstrated that the spatial distributions of animals are influenced by multiple factors, including environmental characteristics, resource availability, interspecific interactions, and human disturbances [16]. For example, environmental changes interact with prey availability to shape the distributions of carnivores [17,18]. Competition for limited resources among sympatric species is often mitigated by partitioning habitats through the utilization of distinct vegetation types [19]. Additionally, human activities, such as road construction, can alter habitat selection and reduce the range sizes of wildlife [20,21]. Synergistic interactions among these drivers affect the spatial distributions of species and highlight the critical importance of integrating multivariate ecological predictors in SDMs to inform targeted conservation strategies [22,23,24,25,26].

The wolverine (*Gulo gulo*), a sentinel species capable of serving as an early warning indicator of broader ecological issues such as biodiversity loss, is broadly distributed across the boreal zone of the Northern Hemisphere. In China, this circum-boreal species is restricted to the cold temperate coniferous forests of the northern Greater Khingan Mountains and the Altai Mountains [27,28]. As a medium-sized apex predator in the northern Greater Khingan Mountains, the wolverine helps maintain regional ecosystem balance by exerting top-down regulatory effects [29]. Its primary prey include moose (*Alces alces*), red deer (*Cervus elaphus*), roe deer (*Capreolus pygargus*), snow hare (*Lepus timidus*), and sable (*Martes zibellina*) [30]. However, because of its naturally low population densities and reproductive output, the wolverine has a small population size and a narrow distribution. Recent research on wolverines has primarily focused on habitat selection [31], interspecific relationships [32], and feeding habits [33]. Kortello et al. employed habitat suitability modeling and genetic analyses to investigate the mechanisms influencing the winter distribution of wolverines in the southern Columbia Mountains of Canada, identifying climate conditions, habitat environment, and human activity disturbances as the primary factors [34]. Ray et al. utilized hierarchical Bayesian occupancy models to assess the distribution patterns of wolverines in remote areas of northern Canada, finding that their distribution is closely associated with topographic complexity, vegetation type, and prey availability [35]. However, critical knowledge gaps remain regarding the spatiotemporal distribution of wolverines and the factors affecting their activity patterns. To address these gaps, we investigated the activity patterns and spatial distribution of wolverines in Beijicun National Nature Reserve, Heilongjiang Province, China, to characterize the ecological characteristics and habitat requirements of this endangered apex predator in this region. Using a multi-method framework that integrates camera-trap data with kernel density estimation and occupancy modeling, we analyzed the spatiotemporal distribution of wolverines and the factors affecting it. We also used MaxEnt to evaluate the effect of spatial distribution on suitable habitat areas and areas of overlap between wolverines and their competitors. We hypothesize that wolverine occupancy will be negatively correlated with high human activity, positively correlated with high prey density, and that wolverines will engage in spatial or temporal avoidance strategies with respect to their competitors.

## 2. Materials and Methods

### 2.1. Study Area

**Figure 1:**
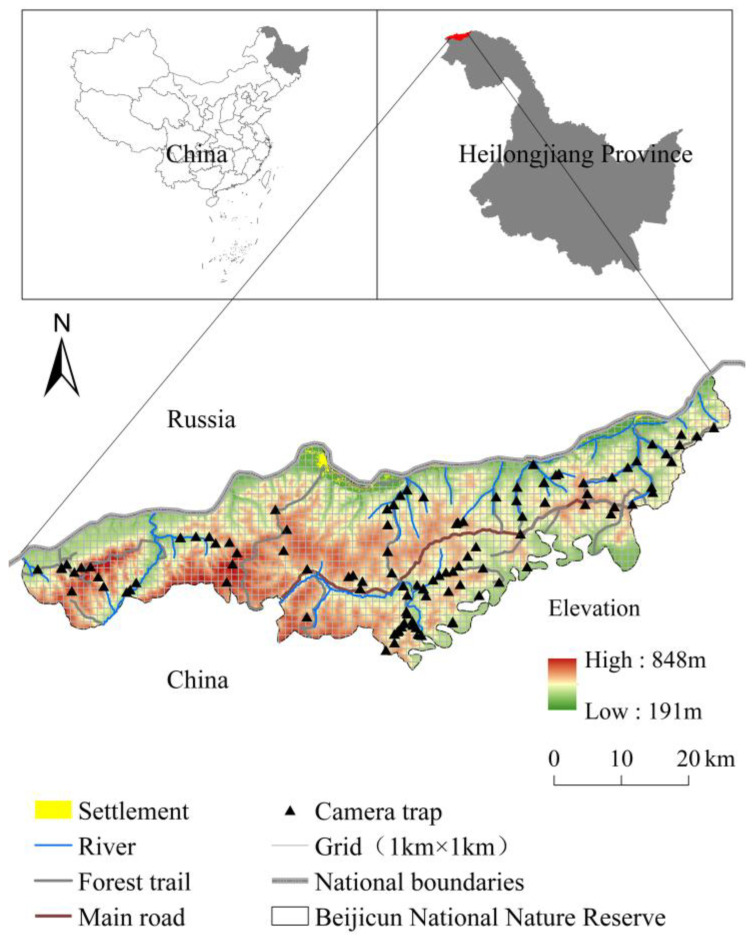
Study area diagram of Beijicun National Nature Reserve.

Beijicun National Nature Reserve is situated in the northern region of the Greater Khingan Mountains in Heilongjiang Province, China, along the southern bank of the Heilongjiang River. It spans a total area of 1375.53 km^2^ (53°11′30″ to 53°33′03″ N, 121°40′00″ to 123°16′00″ E, Figure 1). The reserve experiences a cold temperate continental climate. The mean annual temperature is −5 °C, with the highest average temperature around 18 °C and the lowest average temperature around −30 °C. The extreme minimum temperature in winter can drop to −40 °C. The average annual precipitation is 460.8 mm, and the average frost-free period is 86.2 days. The reserve features rich wildlife resources and is predominantly covered by coniferous forests. Dominant tree species include *Pinus sylvestris* var. *mongolica*, *Larix gmelinii*, and *Betula platyphylla*. As a boreal faunal stronghold, the reserve harbors intact predator–prey guilds including endangered tigers (*Panthera tigris altaica*), Eurasian lynx (*Lynx lynx*), and wolves (*Canis lupus*), along with high-density ungulate assemblages (moose, red deer, roe deer) that sustain apex predator populations.

### 2.2. Data Collection, Covariates, and Modeling Selection

To characterize the spatiotemporal ecology of the boreal wolverine, we implemented a systematic camera-trapping survey (January 2022–April 2023) across Beijicun National Nature Reserve. The study area was divided into 1 km × 1 km grid cells (n = 1376). From these, 140 sampling sites were selected and arranged in the network using a spatial balance design (minimum nearest-neighbor distance ≥ 1 km) (Figure 1). The cameras (UOVision Technology Co., Ltd., Shenzhen, China) were set up at locations with a high probability of animal activity (such as animal trails and near water sources), and covered all vegetation types and the 3 altitude zones (200 m bands) divided according to the altitude range of the study area, in order to maximize the detection probability and spatial representativeness. Positioned approximately 3.5 m from the centerline of these pathways, the cameras were mounted with their horizontal axes about 0.5 m above the path level. Additionally, the GPS coordinates of each camera location were recorded to provide precise geographical information. We programmed the motion-detecting digital cameras to photo + video mode, which captured three photographs when triggered, with a one-second delay between successive image sets. During the monitoring period, cameras underwent quarterly maintenance to replace the battery (Nanping Nanfu Battery Co., Ltd., Nanping, China) and storage card (SD, Sandisk Corporation, Milpitas, CA, USA) to ensure that they operated continuously under extreme temperatures (−45 °C to 30 °C). From 27,874 trap-days, we obtained 7724 valid wildlife photographs of independent detections (mean = 55.2 detections/site); these were filtered via the following standardized protocol: (1) images with vegetation occlusion > 50% coverage were excluded, and (2) images were classified as belonging to independent detections if more than 30 min had elapsed between consecutive photographs of the same species, except when distinct individuals or groups were unambiguously identified within this timeframe. Finally, species identifications and associated data were organized by camera ID in a Wildlife Management Database for subsequent analysis.

To characterize the hierarchical drivers shaping wolverine spatial ecology, we initially generated a dataset with 21 occupancy covariates and 17 habitat suitability predictors on the basis of prior research and field surveys [36,37,38,39]. These data included anthropological disturbance factors, biotic factors (e.g., prey, competitor), and habitat factors (e.g., vegetation type, elevation, slope). All continuous variables underwent Z-score standardization (μ = 0, σ = 1) to minimize discreteness and enhance comparability. Multicollinearity was evaluated via pairwise Pearson correlations, and we only retained covariates with correlation coefficients < 0.5 in subsequent analyses [40]. Next, in the occupancy model simulations, we used decision trees—which screen for uninformative parameters in studies applying model selection with information criteria [41]—to screen out uninformative parameters. This process ultimately retained 9 biologically interpretable occupancy predictors and 2 detection covariates for the simulations (Table 1-M1). Among them, in the biological factors, the infrared camera data revealed negligible detection rates for snow hares (0.56 ± 0.12 detections/day) and small rodents (<0.01 detections/day); therefore, we focused on ungulate prey (moose, red deer, roe deer, wild boar), which represent keystone trophic resources for wolverines [42]. For the habitat modeling analysis, based on ecological requirements, we integrated 14 environmental variables into the MaxEnt model. These included 4 variables shared with occupancy models (distances of forest trails and rivers to camera sites, and elevation, slope) and 10 numerical variables (distances of settlement, main roads, deciduous broadleaf forest, deciduous coniferous forest, evergreen coniferous forest, mixed coniferous-broadleaf forest, wetland, farmland, and grassland to camera sites, and aspect) (Table 1-M2).

### 2.3. Statistical Analysis

#### 2.3.1. Temporal Distribution

To investigate the behavioral plasticity of wolverines in the boreal region with an extreme climate, we employed kernel density estimation to model 24 h activity patterns [43]. Temporal overlap between daily activity patterns of seasons and sympatric species was quantified using the ∆ (∆_4_ for n ≥ 50 detections; ∆_1_ otherwise) following the methodology of Monterroso et al. [44]. Both Δ1 and Δ4 belong to different types of overlap coefficients, but they differ in their calculation logic and application scenarios. Δ1 is an overlap coefficient calculated from density vectors estimated at equally spaced time points, suitable for small sample sizes. Δ4 is an overlap coefficient calculated from density vectors estimated using actual observation time points of species, recommended when both sample sizes are greater than 50 [45]. The value ∆ ranges from 0 to 1; the overlap thresholds were interpreted as follows: ∆ = 0 indicates no overlap between the activity pattern of two seasons or two species, ∆ = 1 indicates complete, ∆ > 0.7 indicates no significant difference, 0.6–0.7 indicates moderate temporal overlap, 0.5–0.6 indicates strong partitioning, and ∆ ≤ 0.5 indicates complete temporal segregation [46]. It is important to note that these categories are intended to provide a structured framework for interpreting the degree of overlap or segregation and should not be interpreted as absolute indicators of ecological interactions in the field. Given the distinct climatic conditions of the study area, seasonal activities were partitioned into the warm season (May–October; mean temp. 8.5 °C) and cold season (November–April; mean temp. −24.3 °C). The following nocturnal periods were defined on the basis of astronomical twilight periods: warm-season nights (19:30–04:00) and cold-season nights (15:30–07:30). Finally, we computed the NRAI using the formula described by Liu et al. [47]:

NRAI = D_i-night_/N_i-total_ × 100 (1)

where D_i-night_ refers to the total number of captures of species i during the night period, and N_i-total_ refers to the total number of captures of this species.

We classified diel activity strategies into six phenotypes on the basis of NRAI thresholds: diurnal (NRAI < 10%), predominantly diurnal (10% ≤ NRAI < 30%), cathemeral (30% ≤ NRAI ≤ 70%), predominantly nocturnal (70% < NRAI < 90%), nocturnal (NRAI ≥ 90%), and crepuscular (NRAI ≈ 50%). These thresholds reflect adaptive responses to boreal light regimes and interspecific competition [48,49]. All analyses were conducted in R 4.3.1 (https://www.R-project.org/ accessed on 30 March 2024) using the overlap package [50] for temporal overlap estimation and the activity package [51] for circular kernel density smoothing [52].

#### 2.3.2. Occupancy Model

To quantify the spatial responses of wolverines to seasonal habitat dynamics, we implemented a single-season, one-species occupancy model framework with an explicit stratification by season (cold season; warm season). Detection histories for wolverines (180 camera workdays: 12 surveys × 15-day sampling occasions) were compiled from November 2022 to April 2023. For each survey period, we recorded wolverine presence or absence on the basis of camera-trap detections. Following the completion of our camera-trapping study, we had sufficient data to model wolverine occupancy. A total of 173 temporally independent events (≥30 min interval threshold) were recorded from 140 independent infrared camera-trap sites. We also assessed wolverine activity patterns by calculating the average capture rate (ACR). This allowed us to identify the seasons when wolverines were most active. Subsequently, we constructed a single-season, single-species occupancy model using the following formula:

ACR = Average (N_ij_/T_j_ × 100) (2)

where N_ij_ denotes the number of independent valid photographs taken by species i in season j, and T_j_ represents the total effective working days of the infrared camera in the season [53].

To characterize the effects of interspecific (sympatric competitor and prey) interactions on wolverine spatial ecology, we quantified resource availability and competitive pressure using the RAI of ungulate prey (moose, red deer, roe deer, wild boar) and sympatric competitors (gray wolves, Eurasian lynx). The RAI value for a single species was calculated as follows:

RAI_s_ = N_i_/D_effort_ × 100) (3)

where N_i_ represents the number of independently valid photographs of the species, with i ranging from 1 to 7, and D_effort_ denotes the total number of trap-days [54]. Independent detections were defined as ≥30 min intervals between conspecific captures (except for social species such as wolves, where pack movements were treated as single events).

To rigorously assess the relative importance of prey, competitors, and human activity on wolverine spatial ecology, we implemented hierarchical occupancy modeling in PRESENCE 5.9 (https://www.mbr-pwrc.usgs.gov/software/presence.shtml accessed on 4 February 2024) [12] and used maximum likelihood estimation to compare all models [55]. Given the combinatorial complexity of multi-species interactions, we structured candidate models through an ecological hypothesis framework: Hypothesis 1: Prey-mediated habitat selection (ungulate RAI-driven occupancy); Hypothesis 2: Competitor avoidance (wolf and lynx RAI-driven spatial and temporal separation); and Hypothesis 3: Anthropogenic avoidance (human activity RAI-driven spatial and temporal separation). We used a priori categorizations of prey (A), competitor (B) species, and human activity in the candidate model, given that multi-species parameterization is overly complex because of the high number of potential combinations of model parameters. Thus, we used single-season, one-species models. Single-species models were prioritized to avoid overparameterization, and model selection was performed on the basis of Akaike’s Information Criterion (AIC). We used AIC rankings to identify the optimal single-species occupancy models for wolverines and explicitly test whether wolverine occupancy was influenced by species A, B, and human activity. The full set of parameters and covariates utilized by the models and their descriptions are provided in Table 1. The model with the lowest AIC value and the highest AIC model weight (AICwt) is the optimal model, and the equivalent model of equal importance is determined using ∆AIC < 2. Covariates with a total AICwt > 0.5 have a significant impact on the distribution of wolverines [56].

#### 2.3.3. Species Distribution Model

We used wolverine occurrence data to map the spatial distribution of habitat suitability for wolverines. We also used occurrence data for prey species (red deer, roe deer, moose) and competitors (wolf, lynx), along with 14 environmental predictors at a 30 m resolution, to explore the effects of prey and competitors on the spatial distribution of wolverines (Table 1). Species occurrence records with a minimum distance of 500 m between presence points were used to mitigate spatial autocorrelation. All raster layers were converted to ASCII format in ArcGIS 10.8 (https://www.esri.com/zh-cn/arcgis/geospatial-platform/overview/ accessed on 27 April 2024) and modeled in MaxEnt 3.4.4 (https://biodiversityinformatics.amnh.org/open_source/maxent/ accessed on 2 June 2024) under the following constraints (Figure S1): (1) 75% of the occurrence points were randomly allocated for model training, and the remaining 25% were used for model validation; (2) model parameters included a maximum of 10,000 background points (BC) and 5000 iterations; and (3) we averaged the outputs of 10 replicate model runs to perform the final habitat suitability assessment for the study area to enhance robustness [57]. Model performance was evaluated using the receiver operating characteristic (ROC) curve, and the area under the curve (AUC) was used as a metric for predictive accuracy [58]. Performance thresholds were as follows: AUC < 0.5 (no predictive power), 0.5–0.7 (moderate), 0.7–0.9 (good), and >0.9 (excellent) [59]. Habitat suitability was classified into three levels using the natural break optimization algorithm in ArcGIS 10.8: (1) not suitable (0–0.14); (2) poorly suitable (0.14–0.43); and (3) highly suitable (0.43–1). We also analyzed the spatial distributions of wolves and lynx using MaxEnt to characterize their spatial overlap with wolverines.

## 3. Results

### 3.1. Climate-Mediated Temporal Pattern Shifts and Temporal Overlap of Wolverines with Prey, Competitors, and Human Activity

**Figure 2:**
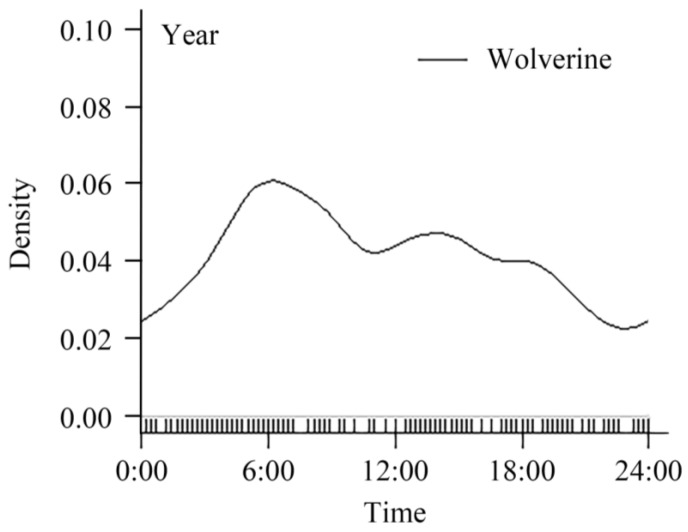
Diel activity patterns of wolverines calculated based on the total days over a 16-month period. The *y*-axis shows the density in activity and the *x*-axis indicates the hour.

Over the 16-month monitoring period, a total of 372 photographic records of wolverines were captured from 140 independent infrared camera-trap sites, yielding 173 temporally independent events. Kernel density estimation for diel activity patterns revealed pronounced bimodal activity, with distinct peaks at 05:00–07:00 (dawn) and 13:00 to 15:00 (early afternoon). The temporal distribution of activity intensity exhibited an “M”-shaped curve, with the highest activity intensity around 06:00 (dawn) and the lowest activity intensity around 23:00 (evening) (Figure 2). The number of independent effective detections in the cold season was 148, whereas in the warm season, it was 25. Among them, only five detections were made during warm-season nights versus 82 in cold-season nights. The total night relative abundance index (NRAI) was 50.29%. These findings suggest that wolverines exhibit crepuscular activity patterns, with peak activity concentrated at dawn and dusk.

**Figure 3:**
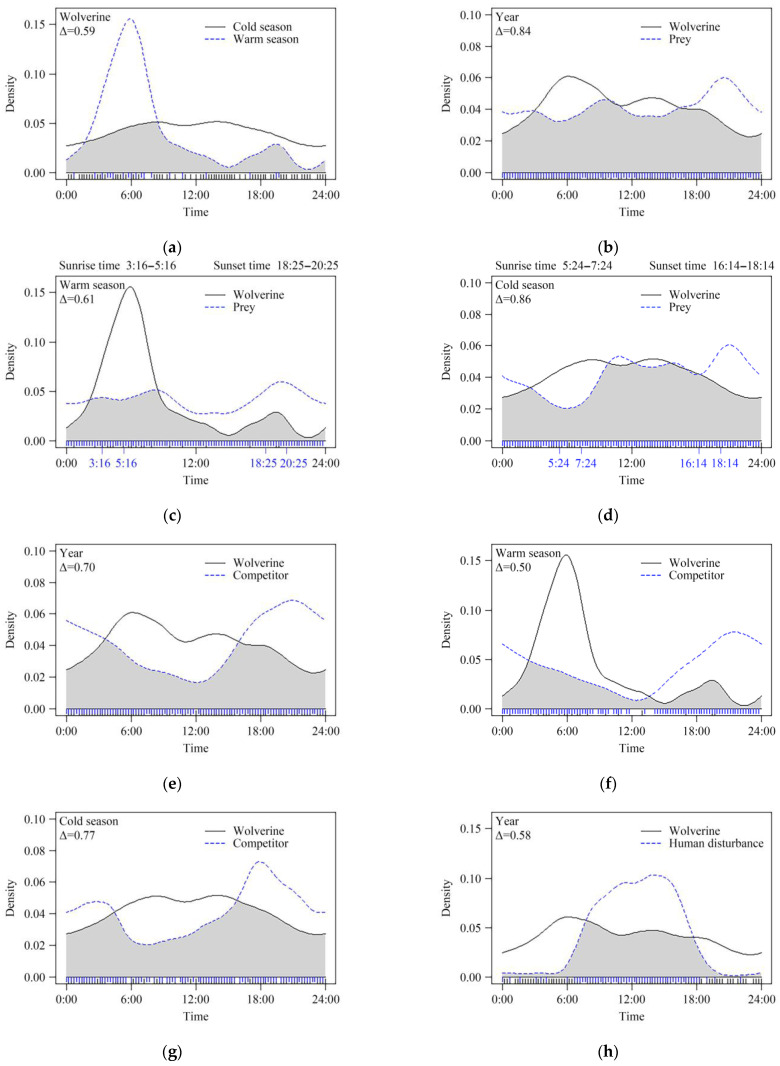
Climate-mediated temporal pattern shifts and temporal overlap of wolverines with their prey, their competitors, and human activity. (**a**) Seasonal variation in wolverine activity patterns between warm and cold seasons; (**b**) diel activity overlap between wolverines and their prey species, calculated based on the total days over a 16-month period; (**c**) overlap between wolverine and prey activity patterns in warm season; (**d**) overlap between wolverine and prey activity patterns in cold season; (**e**) diel activity overlap between wolverines and sympatric competitors, calculated based on the total days over a 16-month period; (**f**) overlap between wolverine and competitor activity patterns in warm season; (**g**) overlap between wolverine and competitor activity patterns in cold season; (**h**) diel activity overlap between wolverines and human disturbance sources, calculated based on the total days over a 16-month period. Overlap coefficients (Δ) are represented by shaded gray areas, with temporal activity patterns of paired groups depicted by solid black (wolverines), and blue dashed (other species/seasons) lines.

Temporal overlap analysis revealed pronounced seasonal divergence in wolverine activity (cold-warm season: Δ = 0.59; Figure 3a). During the warm season, wolverine activity exhibited a unimodal pattern, peaking at dawn (05:00–06:00) and being lowest in the afternoon (15:00–16:00) and at night (21:00–22:00). In cold season, wolverine activity frequency was relatively flat, with higher activity from 08:00 to 09:00 and 14:00 to 15:00, and reduced activity at night (21:00–22:00). Overall, throughout the year, wolverines showed high temporal overlap with their prey (Δ = 0.84; Figure 3b), which were mainly active in the morning (09:00–10:00) and late afternoon (16:00–18:00), peaking at 19:00–20:00 after human activity ceased and being lowest at dawn (05:00–06:00) to avoid threats. Seasonally, warm-season wolverine activity showed moderate divergence from prey activity (Δ = 0.61; Figure 3c), while cold-season activity patterns had high overlap with prey (Δ = 0.86; Figure 3d). Wolverine activity also showed moderate temporal overlap with competitors throughout the year (Δ = 0.70; Figure 3e), reflecting asymmetric avoidance of interference competition, with the highest overlap occurring in the early morning (03:00–04:00) and late afternoon (16:00–18:00). Competitors dominated the nocturnal period, with activity peaking at 20:00–21:00. Seasonally, warm-season overlap with competitors was significantly different (Δ = 0.50; Figure 3f), while cold-season overlap was higher (Δ = 0.77; Figure 3g), indicating that changes in food resources were the main drivers of their activity patterns. Temporal segregation between wolverines and humans was very pronounced (Δ = 0.58; Figure 3h), especially during peak human activity hours during the day (08:00–18:00), with GPS data confirming that 83% of wolverines avoided areas within 1 km of human trails during these hours.

### 3.2. Wolverine Occupancy

Cold-season wolverine detection probability (59.67%) significantly exceeded the annual average (13.59%) in 2022 (mean ratio: 4.39 ± 0.49; *p* < 0.001). Additionally, no correlations were observed among all continuous variables; they could thus be used as predictors in hierarchical occupancy modeling and MaxEnt analyses. The cold-season monitoring period of 2022 included 27,874 trap-days (39/140 sites occupied; grid coverage *ψ* = 0.28). During the simulation of the occupancy models, a total of 14 equivalent models (∆AIC < 2) were obtained (Table 2), among which the optimal model was *ψ* (HUM + A + B + DFT + DBF + DCF + SL + DR), *p*(.).

The optimal model revealed a mean wolverine occupancy probability of *ψ* = 0.35 (SE = 0.086); the most critical factors influencing habitat selection were anthropogenic avoidance elements: human activity intensity (HUM, Sum = 0.67) and distance to forest trails (DFT, Sum = 0.67). Subsequently, prey and competitor-mediated factors played significant roles in habitat selection: prey availability (A, Sum = 0.67) and competitor presence (B, Sum = 0.67). Additional influential factors included forest landscape characteristics: deciduous broadleaf forest (DBF, Sum = 0.67), deciduous coniferous forest (DCF, Sum = 0.67), and topographic constraints, specifically slope (SL, Sum = 0.53).

**Figure 4:**
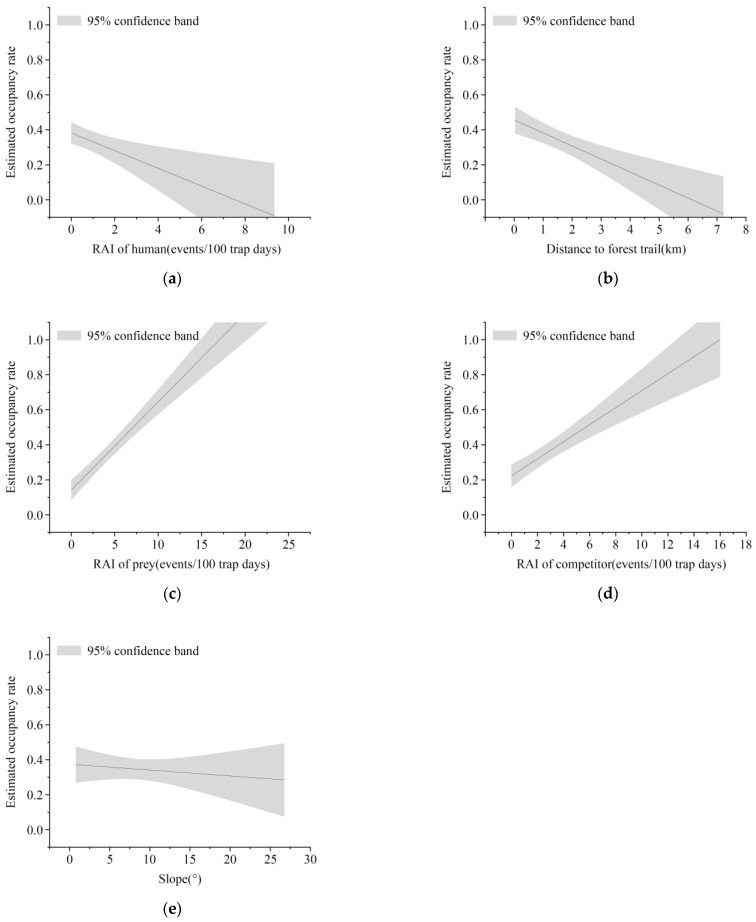
Correlations between wolverine occupancy probability and important covariates in cold season. (**a**–**e**) Correlations of cold-season occupancy probability with the RAI of human, distance to forest paths, the RAI of prey, the RAI of competitor, and slope, respectively.

The corresponding *β* coefficients of each predictor of the optimal model can reflect the extent to which the spatial distribution of wolverine is affected by each predictor (Figure S2). The spatial distribution of wolverines was strongly influenced by human disturbance, and wolverine occupancy was negatively correlated with the relative abundance index of human activity (*β* = −4.02 ± 2.36 SE) (Figure 4a); wolverine occupancy probability decreased with distance from forest trails (*β* = −1.44 ± 0.57 SE) (Figure 4b). Prey resources were also a strong predictor of wolverine activity, and they were positively correlated with wolverine occupancy (*β* = 1.51 ± 0.38 SE) (Figure 4c). However, the presence of competitors did not significantly reduce wolverine occupancy (*β* = 1.04 ± 0.37 SE) (Figure 4d). Wolverines avoided deciduous broadleaf forest (*β* = −2.73 ± 0.82 SE) and deciduous coniferous forest (*β* = −1.77 ± 0.57 SE). Furthermore, slope (*β* = −0.43 ± 0.26 SE) was negatively related to wolverine occupancy, with the probability of occurrence declining steadily as slope increased from 0° to 30° (Figure 4e).

### 3.3. Spatial Distribution Model

**Figure 5:**
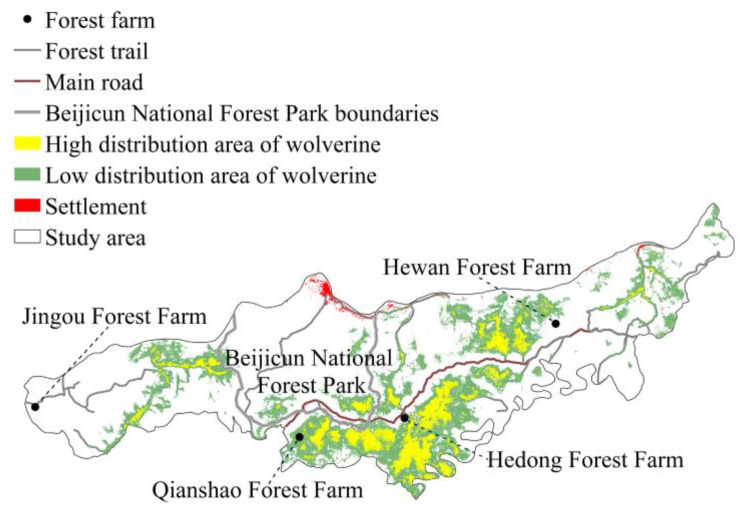
The spatial distribution of wolverines in the Beijicun National Nature Reserve.

The predictive capacity of the MaxEnt model for wolverine distribution was high (AUC = 0.951 ± 0.012; Figure S3), which indicated that the model output provided an accurate reflection of the spatial distribution of wolverines in Beijicun National Nature Reserve. High-suitability habitats (72% of total areas) (probability > 0.43) of wolverines were clustered in Hedong Forest Farm and Qianshao Forest Farm (Figure 5). Although wolverines were detected at other forest farms, the suitability of these areas was low, and the local landscape in these areas is highly fragmented.

**Figure 6:**
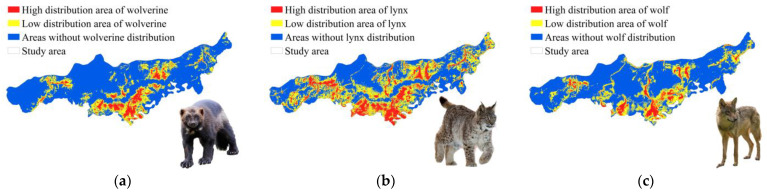
Spatial distribution map of wolverines and competitors (lynxes and wolves). (**a**) Spatial distribution map of wolverines; (**b**) spatial distribution map of lynxes; (**c**) spatial distribution map of wolves.

A map of the spatial distribution of wolverines and their competitors revealed distinct patterns of habitat use (Figure 6). The high-density distribution area for wolverines covered 117.49 km^2^, accounting for 8.54% of the total study area; the low-density distribution area covered 349.08 km^2^ (25.38%), and areas of absence comprised 908.96 km^2^ (66.08%). The high-density distribution area for lynx was 251.90 km^2^ (18.31%), the low-density distribution area was 617.23 km^2^ (44.87%), and areas of absence comprised 506.40 km^2^ (36.82%). The high-density distribution area for wolves was 106.27 km^2^ (7.73%), the low-density distribution area was 416.74 km^2^ (30.29%), and areas of absence comprised 852.52 km^2^ (61.98%).

**Figure 7:**
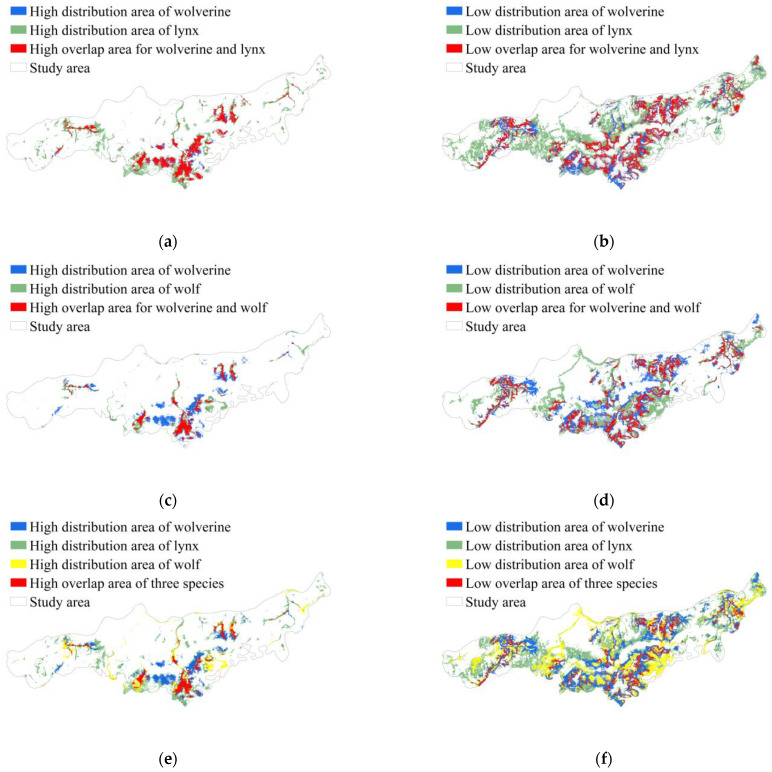
Spatial overlap between wolverines and competitors. (**a**) Overlap area of high distribution between wolverines and lynxes; (**b**) overlap area of low distribution between wolverines and lynxes; (**c**) overlap area of high distribution between wolverines and wolves; (**d**) overlap area of low distribution between wolverines and wolves; (**e**) overlap area of high distribution among the three species; (**f**) overlap area of low distribution among the three species.

Spatial distribution overlap analysis revealed spatial segregation among wolverines, lynx, and wolves. Spatial distribution partitioning in boreal carnivore guilds revealed 86.34% and 41.01% overlap in the high-density distribution of wolverines with that of lynx (Figure 7a) and wolves (Figure 7c), respectively, suggesting that wolverines show extensive spatial overlap with lynx but have more limited spatial overlap with wolves. Moderate overlap was observed in the low-density distribution (lynx: 61.32%; wolves: 46.93%) (Figure 7b,d), indicating facultative avoidance rather than strict territorial exclusion. Notably, the area of overlap in the high-density distributions of all three species was 46.70 km^2^, which constitutes 39.75% of the high-density distribution area of wolverines (Figure 7e).

## 4. Discussion

Temporal patterns of animal activity are influenced by various environmental factors, including individual differences among species, temperature, prey availability, presence of competitors, human activity, vegetation type, elevation, and slope. Seasonal fluctuations in the abundance and accessibility of primary food resources can also drive shifts in activity patterns [60]. In this study, wolverines exhibited a bimodal daily activity pattern, characterized by crepuscular peaks, with activity concentrated during daylight hours (05:00–16:00) and at night (18:00–21:00). Wolverines demonstrated distinct seasonal variations in their daily activities. During the warm season, activity followed a unimodal pattern, peaking at dawn (05:00–06:00). In contrast, during the cold season, activity was more evenly distributed throughout the day (06:00–16:00), and the detection rate was higher. This pattern is consistent with findings reported by Thiel et al. [61], suggesting that wolverines show robust behavioral plasticity across diverse regions and environments.

The study results indicate that the camera-trap detection rate of wolves is significantly higher during the cold season compared to the warm season. This is primarily due to our investigation of the activity patterns of wolverines over a 16-month monitoring period, which included an additional four months designated as the cold season, thereby resulting in a higher detection rate for this period. Furthermore, this pattern may also be related to the following three key factors: (1) Reproductive behavior: The breeding season (January–April) coincides with increased movement rates, as females intensify foraging to meet energetic demands of gestation and lactation [33]. (2) Anthropogenic avoidance: Human activity declines in winter, allowing wolverines to reoccupy high-disturbance zones (e.g., forest trails) that are avoided during peak human-use periods [38]. (3) Resource scarcity: Winter’s reduced prey availability and carcass reliance necessitate broader foraging ranges and more frequent travel to exploit spatially concentrated resources [36].

Our study indicates that the temporal distribution of wolverines reflects a strategic balance between prey resource availability and the avoidance of dominant carnivores. Although the reproductive phenology of prey species—such as concentrated parturition during warm seasons [62]—fundamentally drives detection rates, wolverines demonstrate behavioral plasticity, prioritizing energy acquisition and conflict minimization. They concentrate their activities during the early peak of prey availability (02:00–09:00) while avoiding the peak activity of competitors (21:00), suggesting that predator avoidance may outweigh pure resource optimization—consistent with the “landscape of fear” framework [63,64]. This strategic temporal partitioning probably optimizes energy intake by (1) enhancing encounter rates with prey to reduce capture costs [65], and (2) minimizing interference competition while maximizing access to animal and plant biomass resources. The resulting bimodal activity pattern (with warm-season peaks at 02:00–09:00 and 20:00–21:00) represents an evolutionary adaptation that balances maximizing resource acquisition with reducing the risks of confrontation.

During the cold season, wolverines are mostly active during the daytime, and their activity patterns do not significantly diverge from those of their prey and competitors. This likely stems from several factors. (1) In the cold season, when plant-based food is scarce, wolverines primarily rely on animal-derived resources. This is consistent with optimal foraging theory [66], which predicts that predators should adjust their activity patterns to maximize prey encounter rates. (2) Wolverines also engage in scavenging behavior more during the cold season and spend more time tracking, waiting near, and commuting between large carcasses to secure food. (3) Wolverines exhibited thermoregulatory-driven diel shifts in their activity during winter. Lower night-time temperatures in winter likely force wolverines to remain active during the daytime (06:00–16:00), and their foraging is likely performed in multiple short bouts to reduce energy expenditure. A subtle discrepancy was observed between wolverine and prey activity patterns, which possibly reflects top-down and bottom-up trophic interactions within the food web [67,68]. Predators often seek prey when they are active to enhance foraging efficiency, yet prey may shift their activity to minimize temporal overlap with predators [69]. Similarly, wolverines appear to employ a strategy in which the need to reduce direct competition with dominant scavengers, such as wolves and lynx, is balanced with the need to maximize resource access (Figure 3g); this allows them to exploit shared food sources while mitigating predation risk [70].

Chronic human disturbance drives temporal pattern shifts across wildlife guilds via both fear-mediated behavioral change and reduction in resource abundance, and this has major implications for ecosystem structure and function [71,72,73]. For example, fear-induced range contractions in carnivores may alter the spatiotemporal distribution of predator and thus prey distributions [74]. Recent analyses of wildlife behavior in Southeast Asia have demonstrated that human presence significantly alters temporal activity patterns of wildlife, with some species exhibiting increased nocturnality in disturbed habitats [75]. In our study, our findings show that predators exhibit nocturnal compression. Although human access to Beijicun National Nature Reserve is restricted, seasonal berry harvesting (July–August) represents a major source of anthropogenic disturbance; human disturbances were clustered during the warm season (berry-picking season), correlating with an 83% decline in wolverine detection rates (warm season: 25 independent detections). Wolverines exhibited spatiotemporal distribution change, shifting their activity to crepuscular periods and abandoning 34% of their core foraging zones within 2 km of berry-picking areas. The temporal shift in activity timing (avoiding peak human activity periods) and spatial avoidance of high-risk areas (evading core human-impacted zones) act in concert to form a robust defensive mechanism. This interaction significantly reduces the probability of overlap between wolverines and humans across both temporal and spatial dimensions.

Occupancy modeling results revealed that among anthropogenic disturbance factors, wolverine detection rates were high near forest trails. This pattern may be attributed to (1) the unique geographical setting of Beijicun National Nature Reserve, and (2) effective conservation strategies that have successfully mitigated human impacts on forest trail systems. Additionally, the shrub cover adjacent to forest trails (68 ± 12%) was significantly higher than that near other road types (such as major roads) in the study area (23 ± 8%), supporting more frequent prey activity in these areas. Wolverines preferentially select areas that offer higher energetic returns per foraging effort [76]. Specifically, wolverines would be expected to use these habitats the most when the benefits of prey acquisition outweigh the potential costs, which include both the energy expenditure associated with avoidance behaviors and the risk of injury during encounters with competitors [77]. This finding is consistent with the results of previous studies indicating that wolverines exploit forest trails in minimally disturbed regions [78]. Primary roads, characterized by high traffic volume and frequent human activity, are high-risk areas. Studies have shown that vehicular collisions account for the largest proportion of accidental deaths among wolverines, and survival rates are higher in areas farther from major roads [79]. In response, wolverines exhibit crucial spatiotemporal adaptations: they either accelerate their movement to minimize exposure time on the road or avoid the road entirely [38]. The essence of both strategies is to reduce time spent in hazardous spaces to evade immediate lethal risks. These behaviors collectively demonstrate how wolverines flexibly coordinate their temporal and spatial behaviors based on the nature of disturbances to ensure their own safety.

Wolverines exhibited strong habitat selection for low-gradient slopes (<30°) and lower elevations (400–500 m), where snowpack persistence exceeded 150 days/year. Winter line-transect surveys conducted in the study area revealed that prey encounter rates in these selected habitats were 2.1 times higher than in high-elevation, steep-slope habitats (600–900 m), consistent with the findings of Aubry et al. [31]. Wolverine occupancy rate declined exponentially with the distance from riparian zones; in particular, this pattern intensified during periods when rivers froze (November–March), and the rate of decline increased to 68% per km. This reflects both (1) enhanced mobility via frozen river corridors (movement rates increased 2.3-fold on frozen rivers versus terrestrial routes) [49] and (2) the concentration of ungulate prey, with winter prey densities along riverbanks exceeding those in inland areas. Furthermore, the post-fire homogenization of boreal forests constrains wolverine habitat selection. The 1987 megafire converted primary forests into naturally regenerated secondary forest stands with 92.3 ± 4.7% coniferous coverage (dominated by *Larix gmelinii*) and a relatively uniform understory (*Vaccinium* spp. cover: 78.1 ± 6.2%). The structural heterogeneity of this homogenized landscape decreased by 41%, which effectively eliminated forest-type selection gradients for wolverines.

Apex predators regulate ecosystem stability through both consumptive effects (e.g., direct predation) and non-consumptive effects (e.g., competition, fear-induced behavioral changes from human activity) on prey and competitors [80]. In this study, we examined the spatial distribution of wolverines, as well as the spatial distributions of their potential prey (moose, red deer, roe deer, and wild boar) and two dominant competitors (wolves and lynx) to explore the mechanism by which top predators coexist in the boreal forest ecosystem. Our results indicate that although wolverines exhibit significant spatial overlap with lynx (94.55%) and wolves (65.55%), coexistence may be achieved through fine-scale temporal pattern shifts. Specifically, the activity peaks of wolverines occur during daylight hours (8:00–9:00 and 14:00–15:00), which contrasts with the crepuscular (18:00) and late-night (2:00–4:00) activity peaks of its competitors. This temporal differentiation likely facilitates species coexistence through multiple mechanisms: (1) reducing direct encounters with dominant competitors (e.g., wolves), thereby minimizing interference competition and predation risk; (2) establishing a “temporally delayed” resource utilization pattern, wherein wolverines scavenge carcasses left by nocturnal predators; and (3) conserving energy to enhance survival in cold environments, which was reflected in their more moderate levels of activity during daytime hours. In addition, the observed high spatial overlap may stem from three key factors: (1) as opportunistic predators, wolverines actively track the predatory activities of lynx and wolves to access carrion resources [81,82,83]; (2) wolverines show greater tolerance of intermediate levels of human disturbance than wolves but less tolerance than lynx [84,85]; and (3) a size-mediated hierarchy results in asymmetries in competitive pressure, as social wolves (~50 kg) and solitary lynxes (20–30 kg) exert differing levels of competition on wolverines [32]. Our findings also revealed that temporal pattern displacement mediates interspecific avoidance in boreal carnivore guilds within areas of overlap, with the activity peak of wolverines occurring 3 and 2 h earlier than that of gray wolves and Eurasian lynx, respectively. This temporal partitioning is likely associated with facultative avoidance behaviors. These findings support the “multidimensional niche partitioning” hypothesis, emphasizing that alterations in the spatial and temporal variation of top predators minimize resource competition among them and promote their coexistence [86]. In particular, the differentiation in temporal niches (<3 h) may play a key role in maintaining the stability of predator communities with high spatial overlap [86].

Our findings revealed the combined effects of anthropogenic pressures, trophic resource dynamics, and interspecific competition on the spatial ecology of boreal wolverines. Wolverines primarily utilized the Hedong and Qianshao Forest Farms within the protected area; photographs provided some information on the foraging, food caching, reproduction, and territorial behavior of wolverines. Furthermore, human disturbance is low and prey and competitor densities are high in the southern part of the reserve, as residential areas are concentrated predominantly in the northern part of the reserve. The habitat in the south was classified as suitable for wolverines. By contrast, the northern area of the reserve includes a national forest park, which is a popular tourist destination that experiences a high volume of vehicular traffic. Consequently, much of the northern part of the reserve is unsuitable for sustaining viable wolverine populations.

## 5. Conclusions

Our study applied kernel density estimation, occupancy modeling, and MaxEnt modeling to analyze the spatiotemporal distribution of wolverines in the boreal region of northeastern China. These approaches enabled us to assess how anthropogenic disturbances, interspecific interactions, and environmental variables affect the temporal and spatial distribution of wolverines. Our results indicated that spatiotemporal overlap and partitioning with prey and sympatric competitors facilitated resource access and mitigated interspecific competition. Our research also offers practical guidance for species habitat conservation and management, such as sustainable prey management and minimizing disturbances in high-occupancy areas of wolverines. These findings offer a critical foundation for developing evidence-based conservation strategies for multiple species in the boreal region of northeastern China.

## Figures and Tables

**Table 1:** The final covariates used for constructing the occupancy model and the MaxEnt model.

Covariate	Description	Model Parameter	Species	Model
Human (HRAI)	Relative abundance index (RAI) of human	*ψ*	S1	M1
Forest trail (DFT)	Distance from camera location to forest trail	*ψ*	S1, S2, S3	M1, M2
Prey (A)	RAI of Prey	*ψ*	S1	M1
Competitor (B)	RAI of Competitor	*ψ*	S1	M1
River (DR)	Distance from camera location to river	*ψ*	S1, S2, S3	M1, M2
Deciduous broadleaf forest (DBF)	The vegetation type at the camera location is deciduous broadleaf forest	*p*, *ψ*	S1	M1
Deciduous coniferous forest (DCF)	The vegetation type at the camera location is deciduous coniferous forest	*p*, *ψ*	S1	M1
Elevation (EL)	The elevation at which the infrared camera is located	*ψ*	S1, S2, S3	M1, M2
Slope (SL)	The slope of which the infrared camera is located	*ψ*	S1, S2, S3	M1, M2
Aspect (AS)	The aspect of which the infrared camera is located		S1, S2, S3	M2
Settlement (DS)	Distance from camera location to settlement		S1, S2, S3	M2
Main road (DMR)	Distance from camera location to main road		S1, S2, S3	M2
Deciduous broadleaf forest (DBF2)	Distance from camera location to broadleaved deciduous forest		S1, S2, S3	M2
Deciduous coniferous forest (DCF2)	Distance from camera location to deciduous coniferous forest		S1, S2, S3	M2
Evergreen coniferous forest (ECF)	Distance from camera location to evergreen coniferous forest		S1, S2, S3	M2
Mixed broadleaf–conifer forest (MBCF)	Distance from camera location to mixed broadleaf–conifer forest		S1, S2, S3	M2
Wetland (WET)	Distance from camera location to wetland		S1, S2, S3	M2
Farmland (FA)	Distance from camera location to farmland		S1, S2, S3	M2
Grassland (GR)	Distance from camera location to grassland		S1, S2, S3	M2

Note: *p*: the detection covariates; *ψ*: the occupancy covariates; S1: wolverine; S2: lynx; S3: wolf; M1: occupancy model; M2: MaxEnt model.

**Table 2:** Equivalent model of wolverines in the cold season at Beijicun National Nature Reserve.

Model	AIC	∆AIC	AICwt	No. Par	−2L
*ψ* (HUM + A + B + DFT + DBF + DCF + SL + DR), *p* ( )	539.67	0	0.2213	9	521.67
*ψ* (HUM + A + B + DFT + DBF + DCF + SL + EL), *p* ( )	540.18	0.51	0.1715	9	522.18
*ψ* (HUM + A + B + DFT + DBF + DCF + SL), *p* ( )	540.57	0.90	0.1411	8	524.57
*ψ* (HUM + A + B + DFT + DBF + DCF + DR), *p* ( )	540.65	0.98	0.1356	8	524.65

Note: AIC: Akaike Information Criteria; ∆AIC: difference between each model and the optimal model AIC; AIC wt: AIC model weight; No. Par: number of parameters; −2L: −2 log-likelihood.

## Data Availability

The original contributions presented in this study are included in the article. Further inquiries can be directed to the corresponding authors.

## Supplementary Materials

The following supporting information can be downloaded at https://www.mdpi.com/article/10.3390/biology14091165/s1, Figure S1: Detailed settings of the MaxEnt model; Figure S2: Covariate *β* coefficient of equivalent model for wolverine in cold season; Figure S3: ROC curve of prediction results of the MaxEnt model, and the R code used in this study.
