# Supplementary material for: Analysis of the Effects of Prey, Competitors, and Human Activity on the Spatiotemporal Distribution of the Wolverine (Gulo gulo) in a Boreal Region of Heilongjiang Province, China
Source: Biology (Basel). 2025 Sep 1;14(9):1165. doi: 10.3390/biology14091165 (PMC12467346; doi:10.3390/biology14091165)
Supplement: Supplementary file 1 [file biology-14-01165-s001.zip › Figure S1. Detailed Settings of the MaxEnt model. .pdf]

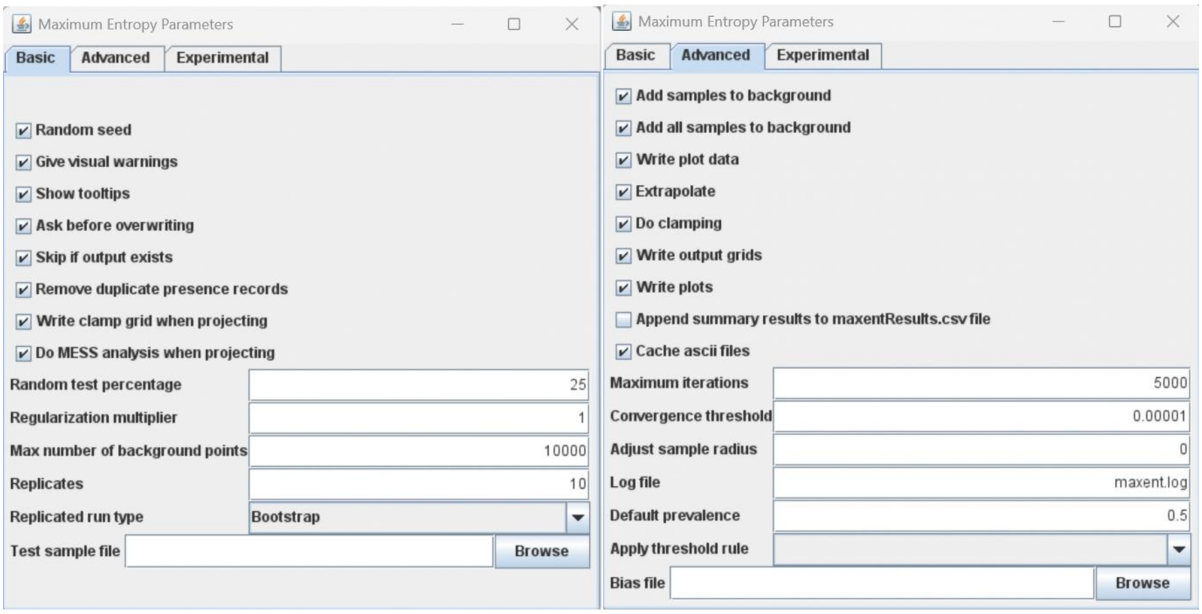

Figure S1. Detailed settings of the MaxEnt model.

Note: Random test percentage: 75% of the occurrence points were randomly allocated for model training, and the remaining 25% were used for model validation; Max number of background points: Model parameters included a maximum of 10,000 background points; Replicates: We averaged the outputs of 10 replicate model runs; Maximum iterations: 5,000 iterations. All other setting parameters are default parameters.
