# Supplementary material for: Analysis of the Effects of Prey, Competitors, and Human Activity on the Spatiotemporal Distribution of the Wolverine (Gulo gulo) in a Boreal Region of Heilongjiang Province, China
Source: Biology (Basel). 2025 Sep 1;14(9):1165. doi: 10.3390/biology14091165 (PMC12467346; doi:10.3390/biology14091165)
Supplement: Supplementary file 1 [file biology-14-01165-s001.zip › Figure S3. ROC curve of prediction results of the MaxEnt model and the R code used in this study.pdf]

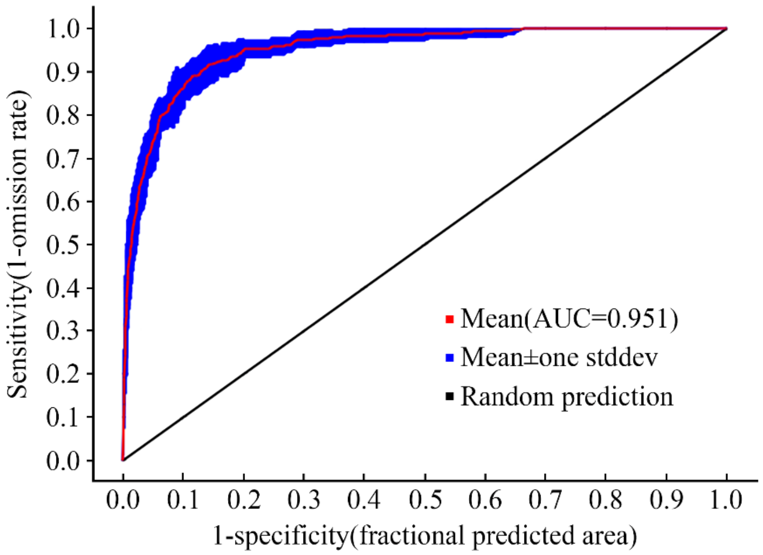

Figure S3. ROC curve of prediction results of the MaxEnt model.

The R code used in this study

```
library(activity)
library(overlap)
library(openxlsx)
getwd()
Qn_version<-read.csv("Qn.csv",header=T,fileEncoding ="GBK")
str(Qn_version)
Qn_version$time<-Qn_version$Photo_time*2*pi
str(Qn_version$time)
Wolverine<-Qn_version$time[Qn_version$Species== "Wolverine"]
windowsFonts(Heiti = windowsFont("Times New Roman"))
densityPlot(Wolverine,rug=T,adjust=1,xlab="Time",
            ylab="Density",lwd=1,main="",ylim=c(0,0.1),family = "Heiti")
legend(12,0.1,c("Wolverine"),col="black",lwd=1,bty="n")


Human_disturbance<-Qn_version$time[Qn_version$Species== "Human disturbance"]
densityPlot(Human_disturbance,rug=T,adjust=1,xlab="Time",
            ylab="Density",lwd=1,main="",ylim=c(0,0.12),family = "Heiti")
legend(12,0.12,c("Human disturbance"),col="black",lwd=1,bty="n")

Prey<-Qn_version$time[Qn_version$Species== "Prey"]
densityPlot(Prey,rug=T,adjust=1,xlab="Time",
            ylab="Density",lwd=1,main="",ylim=c(0,0.1),family = "Heiti")
legend(12,0.1,c("Prey"),col="black",lwd=1,bty="n")
```

```
Competitor<-Qn_version$time[Qn_version$Species== "Competitor"]
densityPlot(Competitor,rug=T,adjust=1,xlab="Time",
            ylab="Density",lwd=1,main="",ylim=c(0,0.1),family = "Heiti")
legend(12,0.1,c("Competitor"),col="black",lwd=1,bty="n")

Qn_version$Species

DX<-as.numeric(Wolverine)
RWGR<-as.numeric(Human_disturbance)
overlapEst(DX,RWGR,type="Dhat4")
fWolverine<-fitact(DX)
f Human_disturbance<-fitact(RWGR)
Loop<-compareCkern(fWolverine,fHuman_disturbance, reps = 1000)
overlapPlot(Wolverine,Human_disturbance, rug=T,main="",xlab="Time",
            ylab="Density",ylim=c(0,0.16),family = "Heiti")
legend(12,0.16, c("Wolverine","Human_disturbance"), lty=c(1,2), col=c(1,4), bty='n')

LW<-as.numeric(Prey)
overlapEst(DX,LW,type="Dhat4")
fPrey<-fitact(LW)
Loop<-compareCkern(fWolverine,fPrey, reps = 1000)
overlapPlot(Wolverine,Prey, rug=T,main="",xlab="Time",
            ylab="Density",ylim=c(0,0.1),family = "Heiti")
legend(12,0.1, c("Wolverine","Prey"), lty=c(1,2), col=c(1,4), bty='n')

JZZ<-as.numeric(Competitor)
overlapEst(DX,JZZ,type="Dhat4")
fCompetitor<-fitact(JZZ)
Loop<-compareCkern(fWolverine,fCompetitor, reps = 1000)
overlapPlot(Wolverine,Competitor, rug=T,main="",xlab="Time",
            ylab="Density",ylim=c(0,0.1),family = "Heiti")
legend(12,0.1, c("Wolverine","Competitor"), lty=c(1,2), col=c(1,4), bty='n')


library(activity)
library(overlap)
library(openxlsx)
getwd()
Ln_version<-read.csv("Wolverine.csv",header=T,fileEncoding ="GBK")
str(Ln_version)
Ln_version$time<-Ln_version$Photo_time*2*pi
str(Ln_version$time)
Wolverine<-Ln_version$time[Ln_version$Species== "Wolverine"]
windowsFonts(Heiti = windowsFont("Times New Roman"))
```

```
densityPlot(Wolverine,rug=T,adjust=1,xlab="Time",
            ylab="Density",lwd=1,main="",ylim=c(0,0.1),family = "Heiti")
legend(12,0.1,c("Wolverine"),col="black",lwd=1,bty="n")

Wolverine_lj<-Ln_version$time[Ln_version$Season== "Cold season"]
densityPlot(Wolverine_lj,rug=T,adjust=1,xlab="Time",
            ylab="Density",lwd=1,main="",ylim=c(0,0.08),family = "Heiti")
legend(16,0.16,c("Wolverine_lj"),col="black",lwd=1,bty="n")
Wolverine_nj<-Ln_version$time[Ln_version$Season== "Warm season"]
densityPlot(Wolverine_nj,rug=T,adjust=1,xlab="Time",
            ylab="Density",lwd=1,main="",ylim=c(0,0.16),family = "Heiti")
legend(16,0.18,c("Wolverine_nj"),col="black",lwd=1,bty="n")

Ln_version$Season

DXLJ<-as.numeric(Wolverine_lj)
DXNJ<-as.numeric(Wolverine_nj)
overlapEst(DXLJ, DXNJ,type="Dhat1")
fWolverine_lj<-fitact(DXLJ)
fWolverine_nj<-fitact(DXNJ)
Loop<-compareCkern(fWolverine_lj,fWolverine_nj, reps = 1000)
overlapPlot(Wolverine_lj,Wolverine_nj, rug=T,main="",xlab="Time",
            ylab="Density",ylim=c(0,0.16),family = "Heiti")
legend(12,0.16, c("Cold season","Warm season"), lty=c(1,2), col=c(1,4), bty='n')


getwd()
Ln_version<-read.csv("Prey.csv",header=T,fileEncoding ="GBK")
str(Ln_version)
Ln_version$time<-Ln_version$Photo_time*2*pi
str(Ln_version$time)
Prey_lj<-Ln_version$time[Ln_version$Season== "Cold season"]
densityPlot(Prey_lj,rug=T,adjust=1,xlab="Time",
            ylab="Density",lwd=1,main="",ylim=c(0,0.08),family = "Heiti")
legend(16,0.16,c("Prey_lj"),col="black",lwd=1,bty="n")
Prey_nj<-Ln_version$time[Ln_version$Season== "Warm season"]
densityPlot(Prey_nj,rug=T,adjust=1,xlab="Time",
            ylab="Density",lwd=1,main="",ylim=c(0,0.16),family = "Heiti")
legend(16,0.18,c("Prey_nj"),col="black",lwd=1,bty="n")

getwd()
Ln_version<-read.csv("Competitor.csv",header=T,fileEncoding ="GBK")
str(Ln_version)
Ln_version$time<-Ln_version$Photo_time*2*pi
```

```
str(Ln_version$time)
Competitor_lj<-Ln_version$time[Ln_version$Season== "Cold season"]
densityPlot(Competitor_lj,rug=T,adjust=1,xlab="Time",
            ylab="Density",lwd=1,main="",ylim=c(0,0.08),family = "Heiti")
legend(16,0.16,c("Competitor_lj"),col="black",lwd=1,bty="n")
Competitor_nj<-Ln_version$time[Ln_version$Season== "Warm season"]
densityPlot(Competitor_nj,rug=T,adjust=1,xlab="Time",
            ylab="Density",lwd=1,main="",ylim=c(0,0.16),family = "Heiti")
legend(16,0.18,c("Competitor_nj"),col="black",lwd=1,bty="n")

Ln_version$Season

LWLJ<-as.numeric(Prey_lj)
overlapEst(LWLJ,DXLJ,type="Dhat4")
fPrey_lj<-fitact(LWLJ)
Loop<-compareCkern(fWolverine_lj,fPrey_lj, reps = 1000)
overlapPlot(Wolverine_lj,Prey_lj, rug=T,main="",xlab="Time",
            ylab="Density",ylim=c(0,0.1),family = "Heiti")
legend(12,0.1, c("Wolverine","Prey"), lty=c(1,2), col=c(1,4), bty='n')

JZZLJ<-as.numeric(Competitor_lj)
overlapEst(DXLJ,JZZLJ,type="Dhat4")
fCompetitor_lj<-fitact(JZZLJ)
Loop<-compareCkern(fWolverine_lj,fCompetitor_lj, reps = 1000)
overlapPlot(Wolverine_lj,Competitor_lj, rug=T,main="",xlab="Time",
            ylab="Density",ylim=c(0,0.1),family = "Heiti")
legend(12,0.1, c("Wolverine","Competitor"), lty=c(1,2), col=c(1,4), bty='n')

LWNJ<-as.numeric(Prey_nj)
overlapEst(DXNJ,LWNJ,type="Dhat1")
fPrey_nj<-fitact(LWNJ)
Loop<-compareCkern(fWolverine_nj,fPrey_nj, reps = 1000)
overlapPlot(Wolverine_nj,Prey_nj, rug=T,main="",xlab="Time",
            ylab="Density",ylim=c(0,0.16),family = "Heiti")
legend(12,0.16, c("Wolverine","Prey"), lty=c(1,2), col=c(1,4), bty='n')

JZZNJ<-as.numeric(Competitor_nj)
overlapEst(DXNJ,JZZNJ,type="Dhat1")
fCompetitor_nj<-fitact(JZZNJ)
Loop<-compareCkern(fWolverine_nj,fCompetitor_nj, reps = 1000)
overlapPlot(Wolverine_nj,Competitor_nj, rug=T,main="",xlab="Time",
            ylab="Density",ylim=c(0,0.16),family = "Heiti")
legend(12,0.16, c("Wolverine","Competitor"), lty=c(1,2), col=c(1,4), bty='n')
```
