# Supplementary figures and images for: Analysis of the Effects of Prey, Competitors, and Human Activity on the Spatiotemporal Distribution of the Wolverine (Gulo gulo) in a Boreal Region of Heilongjiang Province, China
Source: Biology (Basel). 2025 Sep 1;14(9):1165. doi: 10.3390/biology14091165 (PMC12467346; doi:10.3390/biology14091165)

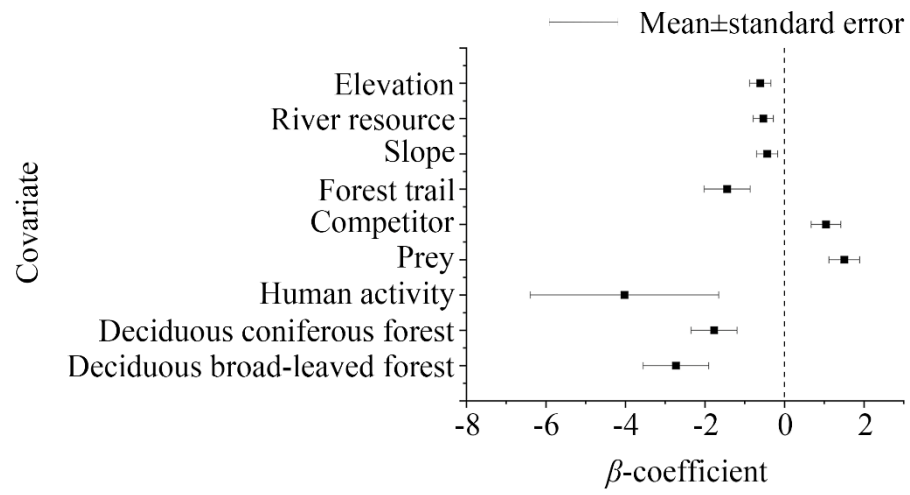

**Figure S2.** Covariate  $\beta$  coefficient of equivalent model for wolverine in cold season.

Supplement: Supplementary file 1 [file biology-14-01165-s001.zip › Figure S2. Covariate aT coefficient of equivalent model for wolverine in cold season..pdf]
